# Reasons for Choosing Dermatology as a Career Choice Among the Dermatology Residents of the Sudan Medical Specialization Board

**DOI:** 10.7759/cureus.88036

**Published:** 2025-07-15

**Authors:** Mahdi Shamad, Yasir Eltahir, Elsheikh Mahgoub

**Affiliations:** 1 College of Medicine, University of Bahri, Khartoum, SDN; 2 Anatomy, Goldfarb School of Nursing, St. Louis, USA; 3 Health Policy, Sudan Medical Specialzation Board, Khartoum, SDN

**Keywords:** career choice, dermatology, future career, residents, sudan medical specialization board

## Abstract

Background and Objectives: Understanding the factors that influence medical doctors' choice of specialty is key to achieving a balanced distribution of healthcare professionals across various medical disciplines. This study aimed to identify the factors influencing the decision to pursue dermatology as a specialty among residents enrolled in the dermatology program at the Sudan Medical Specialization Board (SMSB) (Khartoum, SDN).

Methods: A descriptive, cross-sectional study was conducted among dermatology residents. A predesigned, self-administered questionnaire was distributed electronically to all SMSB-dermatology residents. Data were analyzed using SPSS Statistics version 24 (IBM Corp., Armonk, NY, USA). Descriptive statistics were employed to quantify categorical variables. Chi-square tests were used to compare factors influencing specialty choice. A p-value of <0.05 was considered statistically significant.

Results: There were 313 participants, of whom 304 (97.1%) were female and nine (2.9%) were male. Most (237, 75.7%) were aged between 25 and 35 years, with 236 (75.4%) being married. A large majority (272, 86.9%) chose dermatology after graduation, while 41 (13.1%) decided before graduating. Key factors influencing the choice of dermatology were lifestyle-related, including low stress (284, 90.7%), fewer emergency calls (265, 84.7%), more free time (223, 71.2%), research opportunities (215, 68.7%), financial prospects (209, 66.8%), private sector opportunities (199, 63.6%), the attractiveness of being a dermatologist (198, 63.3%), and procedural opportunities (184, 58.8%). Only 27 participants (8.6%) had a dermatologist in the family, indicating minimal family influence. There was a significant association between gender and subspecialty preference: females were more interested in cosmetology, while males preferred andrology and dermatopathology.

Conclusion: Various factors, as well as both undergraduate and postgraduate dermatology training, influence residents' choice of dermatology as a career.

## Introduction

In recent years, dermatology has become a highly competitive specialty in Sudan, a trend that mirrors developments in many countries both regionally and globally [1]. Since the establishment of the Sudan Medical Specialization Board (SMSB) in 1995, the number of specialties and residency programs has increased, yet dermatology remains one of the most sought-after fields. For instance, in the United Kingdom, competition ratios for dermatology training posts rose from 5.46 in 2021 to 7.53 in 2023 [2].

Dermatology covers various subfields, including sexually transmitted infections, cosmetology, and andrology. Its appeal lies not only in the broad scope of practice but also in the significant advances in medical treatments, which have rendered many chronic skin conditions either curable or more manageable [3]. Moreover, dermatology is largely an outpatient-based specialty, characterized by low on-call demands and flexible working hours, making it particularly attractive to medical graduates [4]. Dermatologists treat patients across all age groups but rarely encounter life-threatening conditions, which contributes to the specialty’s appeal as a low-stress field. Studies from other countries have explored factors influencing doctors' career choices, highlighting key aspects such as income, working hours [5], training duration, patient load, and on-call frequency. In many cases, lifestyle factors, along with doctors' personality traits [6], significantly influence these career decisions. Dermatology is especially popular among female doctors, as it offers a controllable lifestyle with flexible working hours [7]. For instance, in the United Kingdom, 57% of dermatology consultants and 75% of higher specialty trainees in 2016 were women [8].

In Sudan, becoming a dermatologist requires full registration with the Sudan Medical Council. After completing a one-year program post-medical school, which involves rotations in both mandatory and optional specialties, candidates may apply to the SMSB for the Medical Doctorate (MD) in Dermatology and Venereology. The MD program spans four years, including assessments and exams, starting with an entry exam (the 'first part exam') and culminating in an exit exam. In recent years, the number of applicants for the first part exam has surged, often exceeding 600 candidates, with fewer than 100 successfully passing. This heightened competition, influenced by exam difficulty and the limited number of training slots, prompted the core question of this study. The factors that influence Sudanese medical doctors to choose dermatology as their specialty are not well understood. Identifying these factors is important for guiding newly graduated doctors who are considering this career path.

## Materials and methods

Study design

This is a descriptive, cross-sectional study conducted at the SMSB (Khartoum, SDN). The study population comprised residents enrolled in the Dermatology Council at SMSB. These residents were trainees receiving their clinical training at Khartoum Dermatology Teaching Hospitals. All SMSB dermatology residents currently enrolled in the training program who accepted to participate in the study were included.

Sampling method and sample size

Voluntary response sampling by a self‐administered questionnaire was used in this study (see Appendix A). The questionnaire was modified and designed using validated questionnaires from similar studies in other countries [9,10]. To determine the content validity of questionnaire items, a pilot study of a randomly selected sample of 20 SMSB-dermatology residents was conducted using this study’s questionnaire. To improve the reliability of the questionnaire, any inconsistency or confusion in the questions was adjusted. Post-which, the questionnaire was distributed electronically to all SMSB-dermatology residents. The total number of SMSB-dermatology residents enrolled in training at the time of the study was 666, of which 313 responded, with a response rate of 45%.

Data collection

Data were collected using a modified self-administered electronic questionnaire that included both closed- and open-ended questions. The questionnaire comprised sections on demographic information (age, gender, year of graduation, marital status, and parental status) and questions aimed at addressing the research objectives related to specialty choice and factors influencing career decisions.

Data analysis and interpretation

Data were analyzed using SPSS Statistics version 24 (IBM Corp., Armonk, NY, USA). Descriptive statistics were employed to quantify categorical variables. Scores were assigned to questions based on the mean point of all questions in the questionnaire. Chi-square tests were used to compare factors influencing specialty choice. A p-value of <0.05 was considered statistically significant.

Ethical considerations

The study was approved by the Council of Dermatology and Venereology at SMSB. Informed written consent was obtained from all participants, and their privacy and confidentiality were maintained throughout the study.

## Results

Sociodemographic characteristics

The majority (304, 97.1%) of participants were females, while only nine (2.9%) were males. Concerning marital status, 236 (75.4%) of the participants were married, 70 (22.4%) were single, and only seven (2.2%) were divorced. Of the participants, 221 (70.6%) reported having children with variable numbers (Table 1).

**Table 1:** Data on the number of offspring of SMSB dermatology residents who chose dermatology as a career choice SMSB: Sudan Medical Specialization Board

Number of Children	Frequency	Percentage	Valid percentage
None/Not applicable	92	29.4%	29.4%
1 to 2	132	42.2%	(221) 70.6%
3 to 4	73	23.3%	
More than 4	16	5.1%	
Total	313	100%	100%

Graduation background of dermatology residents

Of the total participants, 94 (30%) graduated from three universities, namely the University of Gezira (34, 10.9%), the University of Khartoum (32, 10.2%), and the University of Bahri (28, 9%) (including the former universities of Juba, Upper Nile, and Bahr el Ghazal). These are followed by Omdurman Islamic University (22, 7%) and the University of Kassala (18, 5.8%). The remaining universities were represented by less than 5% (ranging from eight to 15), respectively (Table 2).

**Table 2:** The universities that the SMSB dermatology residents graduated from SMSB: Sudan Medical Specialization Board

Graduation university	Frequency	Percentage	
University of Gezira	34	10.9%	30%
University of Khartoum	32	10.2%	
University of Bahri (including Juba, Upper Nile, Bahr el Ghazal)	28	9%	
Omdurman Islamic University	22	7%	
University of Kassala	18	5.8%	
Elneelain University	15	4.8%	
Al-Zaiem Al-Azhari University	15	4.8%	
University of Red Sea	15	4.8%	
University of Shandi	14	4.5%	
National Ribat University	13	4.2%	
Nile Valley University	12	3.8%	
Ahfad Universality for women	12	3.8%	
International African University	10	3.2%	
University of Kordufan	8	2.6%	
Al-Imam Almahdi Univesity	8	2.6%	
University of Medical Science and Technology	8	2.6%	
University of Gadarif	8	2.6%	
University of Dondola	7	2.2%	
University of Science and Technology	7	2.2%	
University of El Fashir	6	1.9%	
University of Bakht al Ruda	5	1.6%	
University of Sinnar	4	1.3%	
National University	3	1%	
Universities from Abroad (Bucharest; Carol Davila, Romania; Mansoura, Egypt; Aden, Yemen)	3	0.9%	
Al Razi University	2	0.6%	
Ibn Sina University	2	0.6%	
Sudan International University	1	0.3%	
University of West Kordofan	1	0.3%	
Total	313	100%	

The number of graduates kept increasing year on year till it peaked in 2014. A total of 51 (16.3%) participants graduated in the year 2014; 36 graduated in 2013 (11.5%); 33 (10.5%) in 2011; and 32 (10.2%) in 2010. More than 57% (180 residents) graduated between 2010 and 2014 (Table 3).

**Table 3:** Graduation year of SMSB dermatology residents who chose dermatology as a career choice SMSB: Sudan Medical Specialization Board

Graduation year	Frequency	Percentage	Cumulative (percentage)
1994	1	0.3%	10 (3.2%)
2001	2	0.6%	
2003	3	1.0%	
2004	4	1.3%	
2005	18	5.8%	74 (23.6%)
2006	10	3.2%	
2007	13	4.2%	
2008	17	5.4%	
2009	16	5.1%	
2010	32	10.2%	180 (57.5%)
2011	33	10.5%	
2012	28	8.9%	
2013	36	11.5%	
2014	51	16.3%	
2015	26	8.3%	49 (15.7%)
2016	21	6.7%	
2017	1	0.3%	
2018	1	0.3%	
Total	313	100%	313 (100%)

The time of choosing dermatology as a career

**Figure 1:**
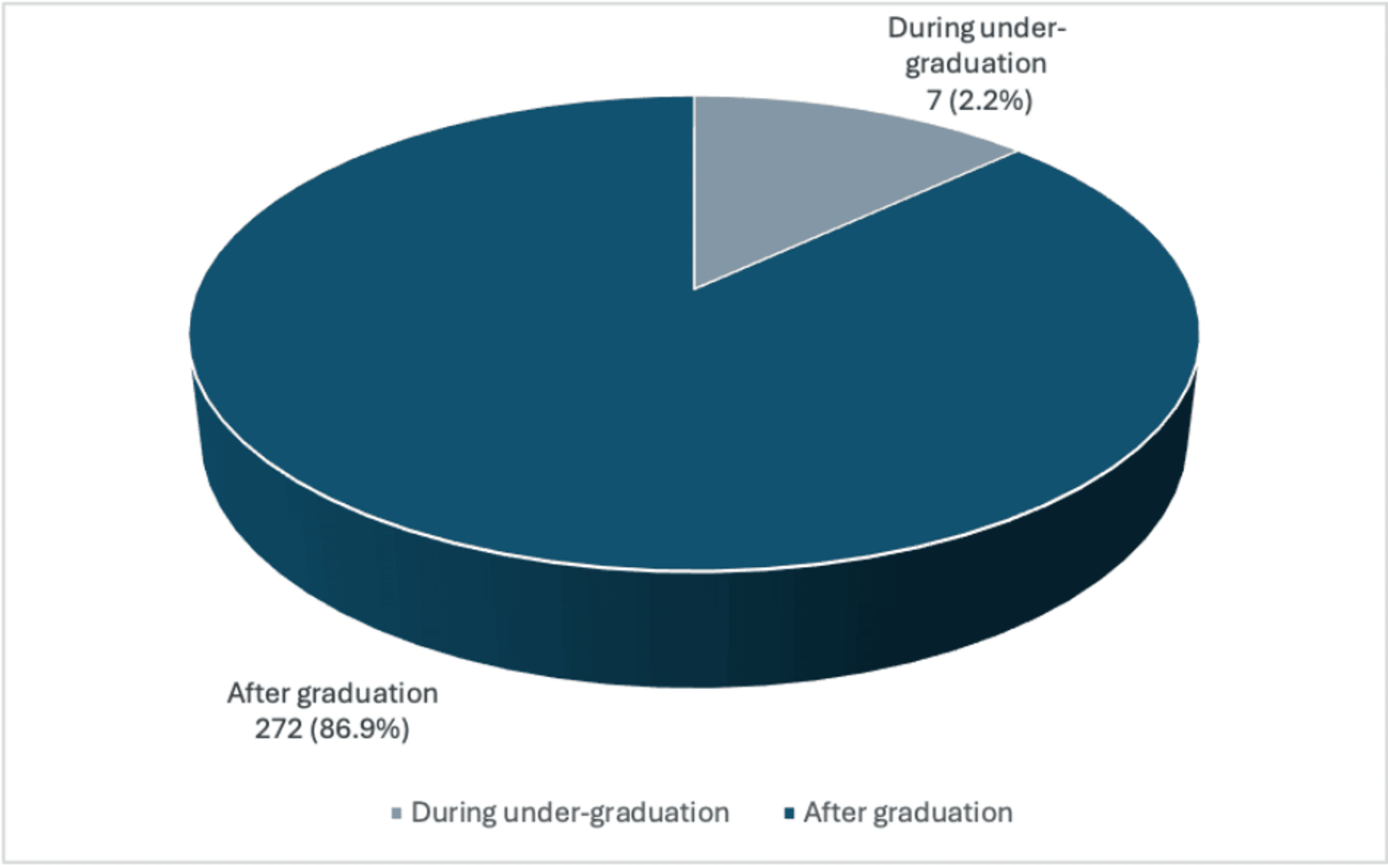
Graph depicting when the SMSB dermatology residents chose dermatology as a career SMSB: Sudan Medical Specialization Board

The vast majority (272, 86.9%) of the participants decided on the specialty after graduation, while 41 (13.1%) opted before graduation (Figure 1).

Factors determining dermatology as a career

Most factors that influenced participants to choose dermatology as a career choice were the low degree of stress (284, 90.7%), fewer emergency calls (265, 84.7%), more free time away from work (223, 71.2%), more opportunities to research dermatology and related subjects (215, 68.7%), good future financial prospects (209, 66.8%), private sector work opportunities (199, 63.6%), attractiveness of being a dermatologist (198, 63.3%), opportunities to perform procedures (184, 58.8%), and high income (175, 55.9%).

Ease of passing dermatology exams (299, 95.5%), affinity for the subject during undergraduate studies (272, 86.9%), personal or family history of skin condition (219, 70.0%), and motivational advice from others (157, 50.2%) were factors that had little to no influence on participants choosing dermatology as a career choice. Less than 50% of participants received motivational advice from others, had a personal or family history of skin conditions, liked the subject during undergraduate studies, and found it easy to pass the dermatology exams (Table 4). The influence of having a dermatologist in the family was also assessed. It was found that only 27 (8.6%) participants have a dermatologist in the family (Table 5).

**Table 4:** Factors that influenced SMSB dermatology residents to choose dermatology as a career choice SMSB: Sudan Medical Specialization Board

Factors		Level of influence	Frequency	Percentage
1	Low degree of stress	No influence	29	9.3%
		Influenced	284	90.7%
2	Fewer emergency calls	No influence	48	15.3%
		Influenced	265	84.7%
3	More free time away from work	No influence	90	28.8%
		Influenced	223	71.2%
4	Opportunities to do research in dermatology	No influence	98	31.3%
		Influenced	215	68.7%
5	Future financial prospects	No influence	104	33.2%
		Influenced	209	66.8%
6	Private sector work opportunities	No influence	114	36.4%
		Influenced	199	63.6%
7	It is attractive to be a dermatologist	No influence	115	36.7%
		Influenced	198	63.3%
8	The opportunity to perform procedures	No influence	129	41.2%
		Influenced	184	58.8%
9	High income	No influence	138	44.1%
		Influenced	175	55.9%
10	Motivational advice from others	No influence	157	50.2%
		Influenced	156	49.8%
11	Personal or family history of skin condition	No influence	219	70.0%
		Influenced	94	30.0%
12	Liked the subject during undergraduate studies	No influence	272	86.9%
		Influenced	41	13.1%
13	Easy to pass the dermatology exams	No influence	299	95.5%
		Influenced	14	4.5%

**Table 5:** SMSB dermatology residents who decided on dermatology as a career choice due to having dermatologists in their family SMSB: Sudan Medical Specialization Board

Dermatologist in family		Frequency	Percent	Total (%)
Yes	Uncle	13	4.2%	27 (8.6%)
	Cousin	5	1.4%	
	Husband’s brother	2	0.6%	
	Father	2	0.6%	
	Grandfather	2	0.6%	
	Sister	2	0.6%	
	brother	1	0.3%	
No		286	91.4%	
Total		313	100%	

Subspecialities of SMSB dermatology residents

According to dermatology subspecialties, most of the participants were interested in skin diseases (142, 54.7%), followed by cosmetology (93, 29.4%), dermatopathology (71, 22.3%), and andrology (seven, 2.6%). A chi-square test of independence revealed a statistically significant association between gender and subspecialty preference (χ² (3, N = 313) = 45.850, p < 0.001; where the degree of freedom is 3, N = sample size). The effect size, measured by phi (φ = 0.383), indicated a medium-strength relationship, suggesting that gender differences play a meaningful role in subspecialty choice. According to these statistical results, there was a statistically significant association between gender and subspecialty preference (p-value = 0.006). Female residents were more likely to prefer cosmetology, whereas male residents showed greater interest in andrology and dermatopathology. From the total of 304 females, 30.6% (n = 93) were interested in cosmetology, and absolutely no males were interested in cosmetology. Males are more interested in andrology (three, 33.3%) and dermatopathology (four, 22.2%) (Table 6). On the other hand, females are less interested in andrology; only four out of 304 females (1.3%) mentioned andrology as the preferred subspecialty. Dermatopathology appeared in the option of 67 females (22%).

**Table 6:** Subspsecialties of SMSB dermatology residents SMSB: Sudan Medical Specialization Board

Subspeciality	Gender				Total	
	Male		Female			
	Number	Percentage	Number	Percentage	Number	Percentage
Dermatology (pure)	2	22.2%	140	46.1%	142	45.4%
Andrology	3	33.3%	4	1.3%	7	2.2%
Cosmetology	0	0%	93	30.6%	93	29.7%
Dermatopathology	4	44.4%	67	22.0%	71	22.7%
Total	9	2.9%	304	97.1%	313	100%

## Discussion

The career selections of medical graduates are crucial to understanding, as they shape the medical labor force, influencing how, where, and when medical care is delivered. Understanding the factors that drive specialty choice is vital for ensuring a balanced distribution of doctors across different fields and planning the healthcare workforce [11]. This study specifically explored dermatology as a career choice, aiming to identify the reasons why dermatology residents selected this specialty.

The sociodemographic characteristics of the participants revealed a significant gender imbalance, with the majority (97.1%) of respondents being female and only 2.9% male. This high proportion of women aligns with previous studies, which indicate that females tend to choose dermatology more often than males, possibly due to the specialty's appeal in both curative and cosmetic aspects. Similar trends have been observed in countries such as Pakistan [12], the US [7], and Japan [13]. Additionally, a study involving over 11,000 medical students from 11 Latin American countries found that the female gender was associated with the choice of dermatology [11]. In contrast, some studies suggest that males tend to select less competitive medical fields [14].

The study found that 30% of dermatology residents graduated from three main universities in Sudan: the University of Gezira, the University of Khartoum, and the University of Bahri, followed by Omdurman Islamic University and the University of Kassala. These universities in Sudan are distinguished by offering a more comprehensive dermatology curriculum. In some cases, dermatology is taught through a dedicated department, with mandatory exams for progression (e.g., the Bahri and Gezira universities). This suggests that the structure of the undergraduate curriculum may have influenced the residents' career choices. The British Association of Dermatologists has also recommended a curriculum with appropriate learning, teaching, and assessment in dermatology based on the modified Delphi technique [15]. This emphasis on dermatology in the curriculum may explain why these universities have produced a higher percentage of dermatology residents compared to others.

The study revealed an increase in dermatology as a career choice in recent years, with over 57% of residents selecting the specialty between 2010 and 2014. This increase reflects a growing trend toward dermatology, which mirrors global trends [9], as the demand for dermatologic services rises alongside aging populations and higher incidences of skin diseases and skin cancer.

The majority of participants (86.9%) decided to pursue dermatology after graduation, while 13.1% made this decision before graduating. Similar findings have been reported in other studies, such as the one by Barat et al., which indicates that dermatologists often make their specialty decisions later in their careers [16]. The key factors influencing residents' choice of dermatology were primarily related to lifestyle [17]. Factors identified in this study, ranked in order, include low levels of stress, fewer emergency calls, more free time, greater research opportunities, and promising financial prospects. These findings are consistent with other studies where lifestyle, flexible work schedules, and personal interest were top factors influencing specialty choice [18,19]. The concept of a 'controllable lifestyle,' defined as control over work hours and time available for activities outside of medical practice, has become a significant factor in choosing a specialty [20]. A similar study in Saudi Arabia found that lifestyle and flexible work schedules were the top two reasons for choosing dermatology as a career [10]. In contrast, in Pakistan, financial prospects ranked as the primary factor, while in the US, a controllable lifestyle remained a strong influence in specialty decisions [21].

Limitations

The study focused on dermatology residents, not comparing career choice influences before and after graduation (e.g., medical students vs. residents). Career choice may change over time due to evolving healthcare demands or economic factors. A comparison with residents from other specialties could have provided deeper insights into whether the observed factors are unique to dermatology or common across medical fields. While demographic comparisons suggest broad representation, further studies are suggested with efforts to boost participation by featuring all residents instead of non-probability.

## Conclusions

Based on the study findings, most residents who chose dermatology as their career are female. The study highlights the growing appeal of dermatology as a career, driven by factors such as lifestyle benefits, flexible work schedules, research opportunities, and the emphasis on dermatology teaching and assessment in medical schools. These findings underscore the importance of considering such factors when planning healthcare workforce strategies, particularly as the demand for dermatologic services continues to rise globally.

## Figures and Tables

**Figure 2:**
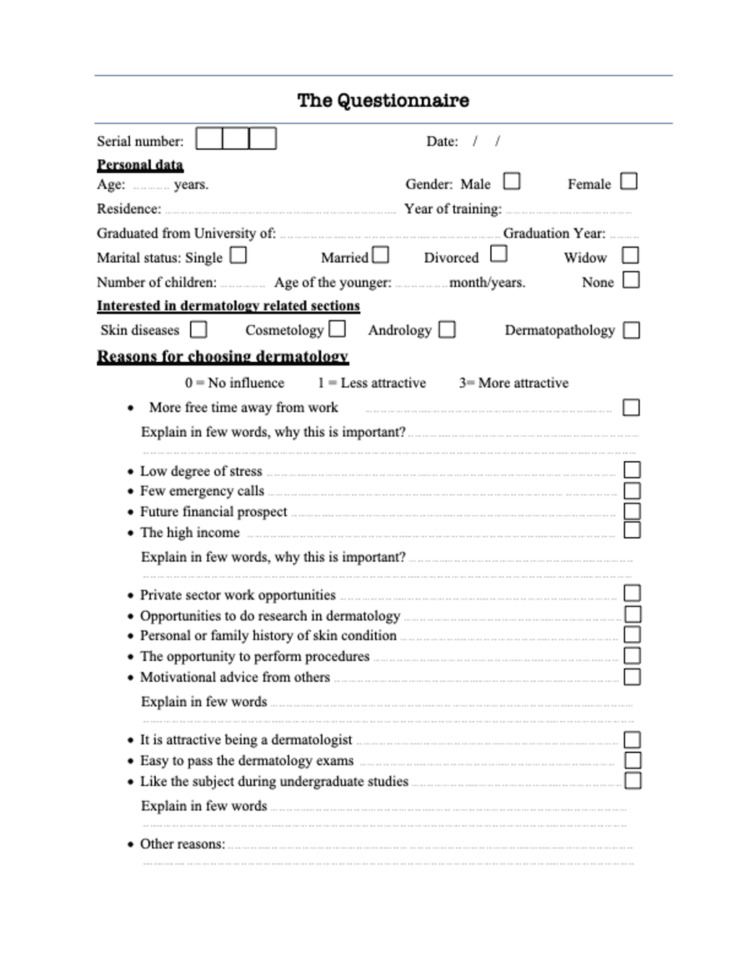
Self-administered questionnaire issued to SMSB dermatology residents SMSB: Sudan Medical Specialization Board

## Appendices

Appendix A

Figure 2 features the modified self-administered electronic questionnaire used to collect data for this study.
